# Methodological rigor of prognostic models for predicting in-hospital paediatric mortality in low- and middle-income countries: a systematic review protocol

**DOI:** 10.12688/wellcomeopenres.15955.1

**Published:** 2020-05-27

**Authors:** Morris Ogero, Rachel Sarguta, Lucas Malla, Jalemba Aluvaala, Ambrose Agweyu, Samuel Akech

**Affiliations:** 1 Health Service Unit, KEMRI / Wellcome Trust Research Programme, Nairobi, P.O Box 43640-00100, Kenya; 2 School of Mathematics, University of Nairobi, Nairobi, P. O. Box 30197 - 00100, Kenya

**Keywords:** Prognostic models, prediction, in-hospital paediatric mortality, model

## Abstract

**Introduction:** In low- and middle-income countries (LMICs) where healthcare resources are often limited, making decisions on appropriate treatment choices is critical in ensuring reduction of paediatric deaths as well as instilling proper utilisation of the already constrained healthcare resources. Well-developed and validated prognostic models can aid in early recognition of potential risks thus contributing to the reduction of mortality rates. The aim of the planned systematic review is to identify and appraise the methodological rigor of multivariable prognostic models predicting in-hospital paediatric mortality in LMIC in order to identify statistical and methodological shortcomings deserving special attention and to identify models for external validation.

**Methods and analysis:** This protocol has followed the guidelines of the Preferred Reporting Items for Systematic Reviews and Meta-Analyses for Protocols. A search of articles will be conducted in MEDLINE, Google Scholar, and CINAHL (via EbscoHost) from inception to 2019 without any language restriction. We will also perform a search in Web of Science to identify additional reports that cite the identified studies. Data will be extracted from relevant articles in accordance with the Cochrane Prognosis Methods’ guidance; the CHecklist for critical Appraisal and data extraction for systematic Reviews of prediction Modelling Studies. Methodological quality assessment will be performed based on prespecified domains of the Prediction study Risk of Bias Assessment Tool.

**Ethics and dissemination: **Ethical permission will not be required as this study will use published data. Findings from this review will be shared through publication in peer-reviewed scientific journals and, presented at conferences. It is our hope that this study will contribute to the development of robust multivariable prognostic models predicting in-hospital paediatric mortality in low- and middle-income countries.

**Registration:** PROSPERO ID CRD42018088599; registered on 13 February 2018.

## Introduction

Despite being readily treatable using cost-effective interventions, malaria, pneumonia, diarrhoea, among others, are the most common conditions attributable to paediatric deaths occurring soon after admission ^1^. Literature has shown that these deaths are inextricably linked to health care related factors ^2^. In low- and middle-income countries (LMICs), evidence-based decision making on appropriate treatment choices is critical in ensuring reduction of paediatric deaths as well as promoting the rational use of constrained healthcare resources. For proper risk selection and initiation of appropriate care and treatment, it is important to be able to predict which patients are at a higher risk of mortality ^3^. To achieve this, clinicians rely on guidelines recommended by the World Health Organization (WHO) detailing a set of simple clinical signs and symptoms for identifying patients at risk of poor outcomes to inform appropriate treatment options ^4^. However, considering that multiple prognostic factors are combined simultaneously when determining patients’ prognosis, clinicians have a challenge quantifying risk. Therefore, prognostic models, which use statistical methods to predict risk levels based on the combination of prognostic factors may improve clinicians’ ability to identify high-risk patients and thus improve outcomes ^5^.

Various clinical prediction models for hospitalized paediatric patients have been developed over time ^6^; however, there are doubts whether appropriate methodology has been used in their development ^7^. Notably, none are currently recommended for use in existing paediatric clinical practice guidelines in LMIC and systematic reviews of the methodology used in the development of these models have been strongly recommended ^8^.

The aim of this systematic review is therefore to address the following questions:

1. Identify and appraise the methodological rigor of multivariable prognostic models predicting in-hospital paediatric mortality in LMIC in order to identify statistical and methodological shortcomings deserving special attention.
2. Identify multivariable prognostic models for external validation.

## Methods and analysis approach

This protocol has adhered to the guidelines and recommended reporting process and checklist outlined in the Preferred Reporting Items for Systematic Reviews and Meta-Analyses for Protocols (PRISMA-P) ^9, 10^. As recommended in the guidelines, this protocol has also been registered with the International Register of Prospective Systematic Reviews (PROSPERO) under registration number CRD42018088599.

## Eligibility criteria

Eligibility criteria for inclusion in the systematic review will be assessed within six domains. Studies will be eligible for inclusion if they meet the criteria for each domain as outlined below:

i) Study design: studies published in peer-reviewed journals and whose design is either a randomized controlled trial, cohort (prospective or retrospective), cross-sectional, or case-control observational study.
ii) Outcome: studies fitting models predicting all-cause in-hospital mortality in a general paediatric ward will be included. Studies predicting post-discharge mortality, trauma or operative mortality will be excluded.
iii) Target population and setting: studies conducted on children aged over 1 month old admitted in general paediatric wards within LMIC as defined by the World Bank ^11^ will be included. Studies whose predictive models targeted uncommon conditions in children, e.g., chronic kidney disease, cancer, and diabetes, will be excluded. Studies conducted on patients in intensive care unit (ICU) or high dependency unit (HDU) will also be excluded because these facilities are largely unavailable in low-resource settings.
iv) Type of study: we will include studies whose main objective is to develop or update clinical multivariable prognostic model in order to predict in-hospital paediatric mortality. We will exclude reports or working papers, commentaries, editorials, expert views, conference proceedings, case reports, case-series, case-reviews and explanatory studies that mainly generate hypothesis.
v) Types of multivariable prognostic models: studies with prognostic models must involve at least two predictors. We will include prognostic models with or without external validation in independent data, and with or without model updating.
vi) Language: non-English language studies will be translated using Google Translate. Hence no language restriction will be enforced.

### Search strategy

As recommended by CHecklist for critical Appraisal and data extraction for systematic Reviews of prediction Modelling Studies (CHARMS) ^12^, we came up with seven key items (see Table 1) applicable to our study that will guide the framing of the search strategy, review, aim and eligibility criteria. We will use Medical Subject Headlines (MeSH) terms with appropriate keywords to identify articles with prognostic studies that match our eligibility criteria (see Table 2). A search of articles will be conducted in the following bibliographic databases: MEDLINE, Google Scholar, and CINAHL (via EbscoHost) from inception to 2019. We will also perform a search in Web of Science to identify additional reports that cite the identified studies. Aware of the potential limitations of electronic search strategies, reference lists of all identified articles will also be searched manually to identify other potentially eligible studies. Final search results will be collated in EndNoteX7™ where duplicates will be removed.

**Table 1.** Review framework according to the CHARMS checklist.

Item	Criteria
Prognostic or diagnostic model	Prognostic model predicting in-hospital mortality.
Scope	Prognostic models to inform clinicians about the risk of deterioration or death.
Type of prediction models	Prognostic models with and/or without external validation.
Prediction target population	Children aged > 1 month to 15 years admitted in paediatric wards in developing countries
Outcome of interest	All-cause in-hospital mortality.
Prediction period	Any
Intended moment to apply the prediction tool	Prognostic model to be used in primary prevention to assess risk of deterioration and thus guide prevention/treatment.

**Table 2.** Search terms.

Search ID	Sub-heading	Search Terms
S4	Children	paediatric* OR pediatric* OR (MH “Paediatrics+”) OR (MH “Pediatrics+”) OR child*
S3	Hospital based	(MH “Hospitals+”) OR hospital*
S2	Low-income countries	(MH “Developing Countries+”) OR (MH “Africa+") OR TI (“low income” OR “low and middle income“OR “LMIC” OR “LIC” OR “limited resource*” OR “poor resource*” OR "resource* poor" OR (“developing countries”) OR (“developing nations”) OR (“third world”) OR “resource-constrained” OR (“global south”)
S1	Predictive models	prognos* OR (MH “prognosis”) OR (Predict* AND (Outcome* OR Risk* OR Model* OR Mortality OR Index OR Rule* OR decision* OR scor*)) OR “risk score” OR “scor* system” OR “logistic model*”“risk prediction” OR “risk calculation” OR “risk assessment” OR “c statistic” OR discrimination OR calibration OR AUC OR “area under the curve” OR “area under the receiver operator characteristic curve”

### Screening of articles and data extraction

We will use a sample of 30 search results to train and familiarize reviewers (MO, LM and JA) with the screening process. Titles and abstracts of the studies from search results will be screened by one reviewer (MO) against the inclusion criteria to select articles for full-text review. A second reviewer (LM) will counter-check the selected articles proposed for inclusion. Should any discrepancy arise regarding extracted data, reviewers will resolve it via discussion and, when necessary, a final decision will be adjudicated by a third reviewer (JA). Reasons will be provided for any articles that shall be excluded from full-text review and the entire process recorded in a flow diagram as stated in PRISMA statement. Data will be extracted from relevant articles in accordance with the guidance of CHARMS checklist.

Box 1 shows the full list of items included in the data extraction form. For articles that describe development of multiple prognostic models, we will treat each model separately if the predictor-outcome association produce different model estimates. For each study, extracted data elements will be compared between two reviewers (MO & LM), and any disagreements will be resolved through discussions with a third reviewer (JA).

Box 1. **Domains and items of the data extraction form.**

*Study characteristics*

- *First Author and year of publication*
- *Model name*
- *Countries where the study was undertaken*
- *Study year (dates when the study was conducted)*
- *Study design/source of data*
- *Patient inclusion criteria*
- *Age distribution of patients*
- *Follow-up period*

*Model development*

- *Sample size*
- *The number of patients with events (deaths)*
- *The strategy used in the selection of the candidate predictors (literature, expert opinion or univariate screening)*
- *Number of candidate predictors considered for modelling/screening*
- *Type of regression model used (Logistic regression or Cox Proportional hazard)*
- *Were model assumptions verified?*
- *If continuous predictors were among the candidate predictors, how were they handled? (categorised/dichotomised, used cubic splines, fractional polynomials or used as is)*
- *Was the multicollinearity among the candidate variables investigated?*
- *How was competing risks handled?*
- *Strategy used to build the final model (forward/backward regression, full model approach) *
- *Number of deaths in relation to the number of candidate predictors in the multivariable model (events per variable)*
- *Total number of predictors included in the final model.*
- *Method used to handle missing data*
- *How were the concerns of overfitting/optimism of the final model addressed?*

*Model Validation*

- *Did authors perform internal validation of the model?*
- *Method used to evaluate the internal validation of the final model (e.g., random split of data, and resampling techniques)*
- *Has the model been externally validated elsewhere?*

*Reporting*

- *Was the extent of missing data per variable reported?*
- *If multicollinearity was investigated, was it reported?*
- *Action taken if multicollinearity was present *
- *List of predictors in the final model, which one of these predictors are laboratory based?*
- *For each predictor included in the final model, was the corresponding regression coefficients/hazard ratios with their confidence intervals, model intercepts or baseline hazard functions reported?*
- *Was the model performance reported both calibration and discrimination?*
- *Was the method used to address model optimism reported?*
- *How is the model reported? Score?*
- *Are classification measures reported (sensitivity, specificity, positive, and negative predictive values)*
- *How is the model presented (e.g., nomogram, score chart, or regression formula with coefficients);*

### Assessment of methodological quality

The risk of bias (shortcomings in the predictive models that might lead to unreliable predictions) of the included studies will be assessed using the Prediction study Risk Of Bias Assessment Tool (PROBAST) ^13, 14^. Risk of bias (RoB) for each model will be assessed in four prespecified domains of the PROBAST: i) participant selection (e.g. study design), ii) predictors (e.g. assessment of candidate and final model predictors), iii) outcome, and iv) analysis (e.g. handling of missing data, competing risks, and the handling of continuous predictors) ( Table 3). For each domain, signalling questions will have five possible answers: yes; probably yes; probably no; no; and no information. RoB in each domain will be judged using the following criteria:

1. Low risk of bias: if all signalling questions are positively answered e.g. yes, or probably yes.
2. High risk of bias: if any of the signalling question is answered as no or probably no.
3. Unclear risk of bias: if the study did not provide adequate information to allow judgement using criteria in 1 and 2 above.

**Table 3.** List of domains and signalling questions used for risk of bias assessment.

Domain	Signalling question
**Participants** **selection**	Were appropriate data sources used, e.g., cohort, RCT, or nested case–control study data?
	Were all inclusions and exclusions of participants appropriate?
**Predictors**	Were predictors defined and assessed in a similar way for all participants?
	Were predictor assessments made without knowledge of outcome data?
	Are all predictors available at the time the model is intended to be used?
**Outcome**	Was the outcome determined appropriately?
	Was a prespecified or standard outcome definition used?
	Were predictors excluded from the outcome definition?
	Was the outcome defined and determined in a similar way for all participants?
	Was the outcome determined without knowledge of predictor information?
	Was the time interval between predictor assessment and outcome determination appropriate?
**Analysis**	Were there a reasonable number of participants with the outcome?
	Were continuous and categorical predictors handled appropriately?
	Were all enrolled participants included in the analysis?
	Were participants with missing data handled appropriately?
	Was selection of predictors based on univariable analysis avoided?
	Were complexities in the data (e.g., censoring, competing risks, sampling of control participants) accounted for appropriately?
	Were relevant model performance measures evaluated appropriately?
	Were model overfitting, underfitting, and optimism in model performance accounted for?
	Do predictors and their assigned weights in the final model correspond to the results from the reported multivariable analysis?

An overall judgement of RoB for each model will be based on the outcomes of the four domains as recommended in PROBAST. For instance, if all four domains in a prediction model will be judged as low, it will be assigned an overall judgment of “low RoB”. If at least one domain in a model will be rated as high, it will be assigned a “high RoB”. Similarly, if at least one domain of the model will be rated as unclear, it will be judged as having an “unclear RoB”.

### Data synthesis

A flow diagram will be used to report the details of the articles screening process indicating reasons for inclusion and exclusion as recommended in the PRISMA statement. Data obtained from each eligible study will be descriptively analysed and summarized by providing tables reporting authors’ names, publication year, study sample and population. For each model reported in the included study, we will narratively synthesize data in terms of candidate predictors, handling of missing data, model development, model performance, evaluation, model presentation, and risk of bias. According to PROBAST, presentation of the risk of bias and assessment of model applicability is an important aspect of communicating the strength of evidence in the systematic review of prognostic models ^14^. Therefore, in this review we will synthesize evidence in terms of the risk of bias on each PROBAST domain and this will be reported as proportions. We will also assess whether meta-analysis is appropriate; if appropriate then random effects meta-analysis of summarizing model performance across included studies will be conducted.

### Strengths and limitation of the study

To our knowledge, this is the first review identifying models predicting in-hospital paediatric mortality in resource-limited settings. Appraisal of the methodological quality of these prognostic models will contribute in identifying statistical and methodological issues that can be potentially improved in developing methodologically sound prognostic models. This will contribute to improving management of patients and accurate stratification of patients for randomised clinical trials. The search strategy used in identifying potential studies in all main electronic databases is robust, hence it is unlikely that a potential study will not be included.

## Conclusion

Appropriate and timely management of common paediatric conditions that contribute to high rates of mortality can be improved through use of well-developed and validated prognostic models that can aid in early recognition of patients with poor prognosis. This is especially critical in resource limited settings. To ensure robustness, models relied upon in predicting hospital mortality for paediatric patients need to have adequate quality. Our findings will potentially be useful in identifying areas for improvements that will go a long way in ensuring appropriate development and description of prognostic models.

### Study status

We confirm that by the time of this protocol submission, article screening had already commenced.

### Ethical approval and dissemination of the findings

For this study, no ethical approval will be required as it will use data from published studies. Findings from this review will be shared through publication in peer-reviewed scientific journals and, presented at conferences.

## Data availability

### Underlying data

No underlying data are associated with this article.

### Reporting guidelines

Harvard Dataverse: PRISMA-P checklist for ‘Methodological rigor of prognostic models for predicting in-hospital paediatric mortality in low- and middle-income countries: a systematic review protocol’. https://doi.org/10.7910/DVN/RKNLGR ^15^.

The completed PRISMA-P checklist is available under the terms of the Creative Commons Zero "No rights reserved" data waiver (CC0 1.0 Public domain dedication).
