# Supplementary material for: Methodological rigor of prognostic models for predicting in-hospital paediatric mortality in low- and middle-income countries: a systematic review protocol
Source: Wellcome Open Res. 2020 May 27;5:106. [Version 1] doi: 10.12688/wellcomeopenres.15955.1 (PMC7364185; doi:10.12688/wellcomeopenres.15955.1)
Supplement: Peer review history

# Peer review: Reviewer response for version 1

**DOI:** 10.21956/wellcomeopenres.17499.r39108

**Published:** 2020-07-15

**Authors:** Charles Opondo

**Affiliations:** 1 University of Oxford, Oxford, UK

This is an exhaustive and well described protocol for a systematic review. I have a few very minor comments:

- In Box 1 where the domains and items of the data extraction form are described, there is need to be more specific about how to document some of the more varied items; for example, how would you appraise a prognostic model that verifies some but not all assumptions; how would you appraise a model that reports some but not all classification measures. These need to be clearly laid out as you have done with other aspects of the protocol.
- Please consider including some kind of scoring of the domains and items; this could form the basis for identifying the statistical and methodological shortcomings in an objective way that may not be achievable through narrative synthesis.

Is the study design appropriate for the research question?

Yes

Is the rationale for, and objectives of, the study clearly described?

Yes

Are sufficient details of the methods provided to allow replication by others?

Yes

Are the datasets clearly presented in a useable and accessible format?

Not applicable

Reviewer Expertise:

Clinical trials, Statistics, Epidemiology, Maternal health, Child health.

I confirm that I have read this submission and believe that I have an appropriate level of expertise to confirm that it is of an acceptable scientific standard.

# Peer review: Reviewer response for version 1

**DOI:** 10.21956/wellcomeopenres.17499.r38868

**Published:** 2020-06-11

**Authors:** James Orwa

**Affiliations:** 1 Medical College of East Africa, Aga Khan University Hospital, Aga Khan University, Nairobi, Kenya

The article on the methodological rigor of prognostic models for predicting in-hospital pediatric mortality in LMICs is a summary of the protocol to be used later in doing a systematic review of the same topic at a later stage. The aims of the protocol are 1) identify and appraise methodological rigor multivariable prognostic models for predicting in-hospital pediatric mortalities and 2) identify prognostic models for external validation.

 The rationale of the study is quantifying the risk of patients risk of poor outcomes which has been set by the World Health Organization based on the multiple prognostic factors. There are also many prognostic models that have been published that pose challenges to clinicians when attempting to quantify the risk. The authors have clearly explained the rationale and all supporting references provided.

 The design of the study is appropriate and is well outlined in the framework (Table 1) and search terms (Table 2) and the process of screening and extracting articles using three reviewers ensures that only published papers that meet the criteria are included in the review.

 Since this is a summary of the protocol, there is no data analysis plan and no data available with this article.

Is the study design appropriate for the research question?

Yes

Is the rationale for, and objectives of, the study clearly described?

Yes

Are sufficient details of the methods provided to allow replication by others?

Yes

Are the datasets clearly presented in a useable and accessible format?

Yes

Reviewer Expertise:

Trained Biostatistician with a major focus on public health research.

I confirm that I have read this submission and believe that I have an appropriate level of expertise to confirm that it is of an acceptable scientific standard.
